# Air pollution and hospital admissions due to deep vein thrombosis (DVT) in Ahvaz, Iran

**DOI:** 10.1016/j.heliyon.2020.e04814

**Published:** 2020-08-28

**Authors:** Seyed Hamid Borsi, Narges Khanjani, Hamid Yazdani Nejad, Atefeh Riahi, Zohreh Sekhavatpour, Hanieh Raji, Maryam Dastoorpoor

**Affiliations:** a Department of Internal Medicine, Air Pollution and Respiratory Diseases Research Center, Ahvaz Jundishapur University of Medical Sciences, Ahvaz, Iran; b Environmental Health Engineering Research Center, Kerman University of Medical Sciences, Kerman, Iran; c Student Research Committee, Ahvaz Jundishapur University of Medical Sciences, Ahvaz, Iran; d Air Pollution and Respiratory Diseases Research Center, Ahvaz Jundishapur University of Medical Sciences, Ahvaz, Iran; e Department of Anesthesiology, School of Paramedicine, Dezful University of Medical Sciences, Dezful, Iran; f Department of Biostatistics and Epidemiology, Air Pollution and Respiratory Diseases Research Center, Ahvaz Jundishapur University of Medical Sciences, Ahvaz, Iran

**Keywords:** Atmospheric science, Environmental assessment, Environmental health, Environmental pollution, Environmental risk assessment, Cardiology, Air pollution, Venous thrombosis, Deep-venous thrombosis, Time-series regression

## Abstract

There are limited studies on the relation between short-term exposure to air pollutants and the risk of deep venous thrombosis (DVT). The aim of this study was to determine the relation between the concentration of these pollutants and the risk of hospital admissions due to DVT in Ahvaz, which is one of the world's highly polluted cities. Daily data on pollutants including O_3_, NO, NO_2_, SO_2_, CO, PM_10_, and PM_2.5_and DVT hospital admissions were collected from2008until 2018. Quasi-Poisson regression combined with linear distributed lag models; adjusted for trend, seasonality, temperature, relative humidity, weekdays, and holidays were used to assess the relation between the daily average of air pollutants and hospital admission for DVT. The results showed that there was a significant increase in hospital admissions due to DVT in the total, men, women, and elderly populations in relation to NO and NO_2_. There was also a significant increase in DVT hospital admissions in the male and ≤60 years populations related to PM_10_; and among the female and ≤60 years old populations, related to PM_2.5_. Finally, the results showed that there were significant positive associations between SO2 and CO exposure and the incidence of DVT hospital admissions among men and women, respectively. The results of this study show the possible effect of short-term exposure to air pollution on the risk of DVT. Further studies are required to investigate whether direct interventions through industry and government policy may alter the impact of specific pollutants in order to alter the incidence of DVT and other identified health complications.

## 1 Introduction

Air pollution is now a global public health problem. Air pollutants include CO, SO_2_, NO_2_, O_3_, nitrate, sulfate, organic compounds, and particles (Tang et al., 2016).

Several studies conducted in Iran have shown that air pollutants can increase cardiovascularmorbidity and death (Dastoorpoor et al., 2018; Dastoorpoor, Sekhavatpour, et al., 2019; Vahedian et al., 2017). These adverse consequences may bedue to the effect of air pollutants on the blood coagulation process, which hasnegative effectson blood circulation (Dales et al., 2010). According to the American Heart Association (AHA) report, ambientparticulate mattermay havethrombotic effectsthrough mechanisms that are not yet well defined (Brook et al., 2010).

Although previous researchhas shown that common risk factors such as age, obesity, diabetes, high blood pressure, and hyperlipemia contribute to the formation of thromboembolism, recent evidence suggests that exposure to air pollution can contribute to lung inflammation and blood coagulation, which helps clarify the link between exposure to air pollution and thromboembolism (Chen et al., 2017; Kloog et al., 2015).

Venous thromboembolism (VTE) involves deep vein thrombosis (DVT) and pulmonary embolism (PE). Deep vein thrombosis (DVT) occurs in the deep veins of legs and thighs. Theclot can stop blood flow, or break apart and move to the lungs, and cause pulmonary embolism (PE). VTE is a multi-factorial disease with serious short and long-term complications. It is potentially fatal and requires immediate medical care. VTEis the third leading cause of death after heart attacks and stroke in the United States, andmight be a contributing cause for the death of 300,000 to 600,000 people annually (Benjamin et al., 2017). In Iran, there is no national registry for recording VTE cases. However, some researchers have estimated the average annual number of total adult patients with predisposingconditions for DVT to be around 5,288,272 people, themean annual frequency of DVT between 686,928 and 2,089,738cases; andthe mean annual prevalence rate of DVT among hospitalized Iranianadult patients, with the risk of DVT to be between 129.90 and395.16 cases per 1000 patients (Sharif-Kashani et al., 2015).

The factors that can cause DVT include pregnancy, the use of contraceptives, and the use of estrogen hormones to reduce the symptoms of menopause (Benjamin et al., 2017). However, according to some recent studies exposure to polluted air (Baccarelli et al., 2008; Dales et al., 2010; Kloog et al., 2015; Signorelli et al., 2017)might be the cause of DVT as well.

NO can affect the respiratory system and cause inflammation in the lungs, and secondary systemic effects on the cardiovascular system, through blood circulation; and can cause atherosclerosis by its adverse effects on the platelets, vascular tissues and myocardium (Spiezia et al., 2014).

Ambient air particles may interfere with pro-coagulants, cause inflammation in the arteries, and have negative effects on platelets, coagulation factors, inflammatory markers, and endothelial markers (Signorelli et al., 2017). Other animal and human studies have shown that exposure to PM can increase hemostasis, release of histamine, and the formation of thrombosis. Air pollution increases levels of homocysteine in plasma, which is a known risk factor for arterial and venous thrombosis (Tang et al., 2016). SO_2_can cause cardiovascular disease (Bhandarkar, 2013; Greenberg et al., 2016; Mabahwi et al., 2014), andthe hypoxia caused by CO results in inadequate sensory and muscle functions in the brain, heart, walls of blood vessels and platelets (Greenberg et al., 2016; Mabahwi et al., 2014; Wark and Warner, 1998).

Baccarelli et al. in a study on 870 people (1995–2005) in Italy showed that there was a significant relation between ambient PM_10_ and DVT (Baccarelli et al., 2008). Another study from Italy showedthe risk of DVT increased in subjectsliving near a major traffic road (3 m) compared to thoseliving further away (245 m) (Baccarelli et al., 2009). Dales et al. (2010) showed thatincrease in ozone, sulfur dioxide, nitro dioxide, and PM_2.5_ increased the incidence of DVT in Chile (Dales et al., 2010). Finally, a study done by Kloog et al. in North America during 2000–2008, found that short-term exposure to particulate matter (PM) had a directeffect on the number of visits to the emergency department due to DVT (Kloog et al., 2015). According to a World Health Organization reporton 1100 cities from91 countries, during 2003–2010, the highest average daily concentration of PM_10_ was seeninAhvaz, Iran (372 μg/m^3^) and Ulan Bator, Mongolia (279 μg/m^3^) (Cohen et al., 2005).

There are a limited number of studies aboutthe relation between air pollutants and DVT. Some studies have only evaluated one air pollutant (Baccarelli et al., 2008; Kloog et al., 2015)or have used proxies for air pollution such as distance from road traffic (Baccarelli et al., 2009). Despite the significant association reported in several studies (Baccarelli et al., 2008, 2009; Dales et al., 2010; Kloog et al., 2015), a recent review denied the relation between air pollution and venous thrombosis (Tang et al., 2016). These controversies highlight the necessity of doing more research.

A study about air pollution and DVT has not been done before in Ahvaz, although it is one of the most polluted cities in the world. The purpose of this study was to determine the relation between air pollutants and therisk of hospital admissions due to DVT, in Ahvaz, Iran.

## 2 Materials and methods

This study was a time series, ecological study. Anonymous data on hospital admissions due to DVT from March 20, 2008 until March 21, 2018 (10 years) were obtained from two main referral hospitals in Ahwaz, which were the Imam Khomeini Hospital and Golestan Hospital.

The daily concentrations of air pollutants in Ahvaz during the 10-year study period were acquired from the Environmental Protection Agency of Khuzestan province. These air pollutants were carbon monoxide (CO), nitric oxide (NO), nitrogen dioxide (NO_2_), particulate matter with a diameter of less than 10μm (PM_10_), particulate matter with a diameter of less than 2.5 μm (PM_2.5_), sulfur dioxide (SO_2_), and ozone (O_3_). Volatile organic compoundswere not included, because they are not routinely measured in Ahwaz.

In Ahvaz, in four points which are Naderi Square, the Khuzestan Environment Department, Ahvaz Chamran University and Khuzestan Meteorological Department, stations for measuring ambient air pollutants have been installed. These stations measure ambient air pollutants and particulate matter less than 2.5 and 10 microns separately and in completely different ways.

For measuring particulate matter, initially air enters the measuring devices by pumps. Then the device measures particle concentration based on absorption intensity, and records the concentration every hour, and stores the filters.

In order to measure other gases, the air sucked in enters different devices separately. After measuring the amount of pollutants, the concentrations are recorded on the servers and then the information is sent to the center. At the monitoring center, all information is recorded and analyzed, and what is declared as the quality indicator is the average of 24 h, which is announced publicly.

The average daily temperature and relative humidity values were acquired from the Meteorological Office of Khuzestan province.

The city of Ahvaz with a total area of 185 km^2^ is the capital of Khuzestan province in Iran. Ahvaz is located at 48º44′39″E, and 31º20′41″Non the plains of Khuzestan province. According to the 2011 census, the population of this city was 1,112,021 people (Statistics").

Missing data was estimated using the Expectation Maximization (EM) algorithm; this algorithm is considered by some references as the most accurate method for estimating missing data (Afshari Safavi et al., 2015).

The relation between daily concentrations of air pollutantsand the number of admissions due to DVT were estimated using Generalized Linear Models (GLM) withDistributed Lag Models (DLM). A quasi-Poisson model was used for the GLM due to the count nature of the dependent variable. Air pollution may have lagged effects that have to be taken into account. In this study DLM was used to assess the effects of air pollutants for up to 7 days. Lag terms were modeled separately, and then all together in unconstrained and constrained adjusted models. A natural cubic spline with 7 degrees of freedom (df) per year was used to control seasonality and the long-term trend (Bhaskaran et al., 2013). The effect of relative temperature and humidity was adjusted by a natural cubic spline function with 6 and 3 degrees of freedom respectively (Bhaskaran et al., 2013; Phung et al., 2016; Ye et al., 2016). The degrees of freedomweredetermined by minimizing the Akaike's Information Criterion (AIC). Similar studies have all used a degree of freedom below 10. In this study, a df range of 1–10 was tested, and the degree of freedom inthe model with the lowest AIC for each variable (seasonality, long-term trend, temperature, and relative humidity) was selected. The model was also adjusted for the effect of days of the week and holidays. A separate model was ultimately developed for each pollutant, because of the collinearity between pollutants (Phung et al., 2016). The analysis was performed in R-software, version 3.3.1, using time-series analysis with theDLNM (Distributed Lag Non-linear Models) package (14). The level of statistical significance was set at P < 0.05 in this study. This statistical approach has been used in similar previous studies (Dastoorpoor, Masoumi, et al., 2019; Dastoorpoor, Sekhavatpour, et al., 2019).

### 2.1 Ethics approval and consent to participate

We assured them that their information would remain confidential. Ethics License of the present study was acquired from the Ethics Committee of Ahvaz Jundishapur University of Medical Sciences (Code of ethics: IR. AJUMS.REC.1397.641).

## 3 Results

Over the 10-year study period, there were 2526 DVT hospital admissions (51% male; 55% among individuals ≤60 years; mean admissions per day = 0.7 ± 1.5). The male to female ratio was 1.36. The average daily concentration of PM_10_ and PM _2.5_were216.9 and 85.7 μg/m^3^, respectively, which was abovethe24-hour standard threshold of the US Environmental Protection Agency (150 and 65 μg/m^3^). A descriptive summary of the variables used in this study can be seen in Table 1.

**Table 1:** The number of hospital admissions due to DVT in population subgroups and descriptive statistics of air pollutants and meteorological parameters in Ahvaz city, 2008–2018.

Variable (Mean per day)	N	Mean	SD	Min	Max	Q1	Median	Q3
Total people	2526	0.7	1.5	0	14	0	0	0
Men	1280	0.4	1.0	0	12	0	0	0
Women	1246	0.3	1.0	0	10	0	0	0
≤60years	1389	0.4	1.0	0	8	0	0	0
>60 years	1137	0.3	1.0	0	10	0	0	0
O_3_ (μg/m^3^)	---	70.5	188.6	0.04	6520.0	26.4	42.7	64.6
PM_2.5_ (μg/m^3^)	---	85.7	150.4	0.57	3938	35.9	52.7	81.2
PM_10_ (μg/m^3^)	---	216.9	278.3	1.8	4324.2	105.8	149.2	222.4
NO_2_ (μg/m^3^)	---	46.4	43.1	1.5	443.8	17.8	35.6	60.7
NO(μg/m^3^)	---	29.1	31.0	0.12	608.7	9.1	19.6	38.2
CO (μg/m^3^)	---	1.5	2.1	0.1	70.4	0.6	1.13	2.0
SO_2_ (μg/m^3^)	---	48.8	57.0	0.0	907.4	19.3	35.9	59.3
Temperature (° C)	---	27.0	9.4	1.4	47.8	18.4	27.7	36.0
Relative Humidity (%)	---	42.3	17.9	7.0	96.0	27.5	40.0	54.5

Tables 2to 4 show the relation between air pollutants and hospital admissions due to DVT in the totalpopulation, andin gender and age groups, up to 7-day lags after exposure, adjustedfor long-term trend, weather parameters, weekdays, and weekends for eachpollutant separately.

There was a significant relation between NO and hospital admissions due to DVT in the men, women (Table 3), and people >60 years of age (Table 4); and for each 10 μg/m^3^ increase in NO, the risk of DVT hospital admissions among men, women and >60 years, increases by 2.7, 3.2, and 2.7 percent in adjusted unconstrained modelson 0, 5, and 1 day lags (Tables 2, 3, and 4).

**Table 2:** The increased risk (95% CIs) of DVT hospital admissions per 10 μg/m^3^ increase in air pollutants in total.

Lag	Pollutant	Total people		
		Lag terms modeled one at a time RR (risk ratio) (95% CI)	Adjusted unconstrained DLM RR (95% CI)	Adjusted constrained DLM RR (95% CI)
Lag 0	O_3_	0.992 (0.983–1.001)	0.989 (0.980–0.999)	0.991 (0.983–1.000)
	NO	**1.002 (1.001–1.004)**	1.013 (0.996–1.031)	1.016 (0.999–1.032)
	NO_2_	**1.037 (1.018–1.055)**	**1.028 (1.003–1.053)**	**1.030 (1.008–1.053)**
	SO_2_	1.005 (0.982–1.029)	1.007 (0.980–1.034)	1.006 (0.981–1.032)
	CO	1.324 (0.937–1.873)	1.339 (0.891–2.012)	1.277 (0.885–1.843)
	PM_10_	1.000 (0.997–1.002)	1.000 (0.997–1.002)	1.000 (0.997–1.002)
	PM_2.5_	**1.003 (1.000–1.005)**	**1.002 (1.000–1.005)**	**1.002 (1.000–1.005)**
Lag 1	O_3_	1.001 (0.997–1.004)	1.007 (0.998–1.015)	**1.001 (1.000–1.003)**
	NO	**1.002 (1.000–1.003)**	1.014 (0.995–1.034)	1.005 (0.995–1.016)
	NO_2_	0.999 (0.985–1.013)	1.008 (0.978–1.039)	**1.020 (1.000–1.039)**
	SO_2_	0.996 (0.971–1.020)	0.991 (0.959–1.024)	0.994 (0.977–1.010)
	CO	1.184 (0.787–1.783)	1.044 (0.640–1.704)	1.124 (0.861–1.468)
	PM_10_	1.000 (0.997–1.002)	1.001 (0.998–1.003)	1.000 (0.998–1.001)
	PM_2.5_	1.001 (0.998–1.004)	1.001 (0.997–1.004)	1.000 (0.998–1.002)
Lag 2	O_3_	1.001 (0.997–1.005)	0.996 (0.988–1.005)	**1.001 (1.000–1.003)**
	NO	1.000 (0.998–1.002)	1.004 (0.983–1.026)	1.005 (0.995–1.016)
	NO_2_	0.999 (0.985–1.013)	1.001 (0.970–1.033)	0.997 (0.977–1.017)
	SO_2_	0.992 (0.967–1.017)	0.997 (0.964–1.031)	0.994 (0.977–1.010)
	CO	1.177 (0.777–1.781)	1.292 (0.791–2.110)	1.124 (0.861–1.468)
	PM_10_	0.998 (0.996–1.001)	0.999 (0.996–1.002)	1.000 (0.998–1.001)
	PM_2.5_	1.001 (0.998–1.003)	1.000 (0.997–1.004)	1.000 (0.998–1.002)
Lag 3	O_3_	0.995 (0.988–1.003)	0.999 (0.988–1.009)	0.999 (0.997–1.000)
	NO	0.998 (0.996–1.000)	0.975 (0.952–0.998)	1.000 (0.993–1.006)
	NO_2_	1.001 (0.995–1.007)	0.973 (0.942–1.006)	0.986 (0.966–1.007)
	SO_2_	0.993 (0.968–1.018)	0.998 (0.965–1.032)	1.002 (0.994–1.009)
	CO	0.902 (0.494–1.645)	0.746 (0.389–1.431)	0.969 (0.796–1.178)
	PM_10_	0.995 (0.992–0.999)	0.995 (0.991–0.999)	1.000 (0.999–1.001)
	PM_2.5_	0.998 (0.995–1.002)	0.999 (0.995–1.002)	0.999 (0.998–1.001)
Lag 4	O_3_	0.993 (0.985–1.002)	0.995 (0.985–1.005)	0.999 (0.997–1.000)
	NO	1.000 (0.998–1.002)	1.013 (0.992–1.035)	1.000 (0.993–1.006)
	NO_2_	1.001 (0.995–1.007)	1.020 (0.988–1.053)	1.001 (0.981–1.022)
	SO_2_	0.999 (0.975–1.024)	1.005 (0.974–1.038)	1.002 (0.994–1.009)
	CO	1.106 (0.698–1.75)	1.075 (0.629–1.838)	0.969 (0.796–1.178)
	PM_10_	0.998 (0.996–1.001)	0.999 (0.996–1.002)	1.000 (0.999–1.001)
	PM_2.5_	0.999 (0.996–1.002)	0.999 (0.995–1.003)	0.999 (0.998–1.001)
Lag 5	O_3_	0.998 (0.992–1.003)	1.000 (0.992–1.009)	0.999 (0.997–1.000)
	NO	1.000 (0.998–1.002)	0.999 (0.978–1.021)	1.000 (0.993–1.006)
	NO_2_	1.001 (0.995–1.007)	0.989 (0.959–1.020)	1.008 (0.988–1.029)
	SO_2_	1.003 (0.980–1.028)	0.992 (0.960–1.025)	1.002 (0.994–1.009)
	CO	1.146 (0.739–1.776)	1.334 (0.720–2.473)	0.969 (0.796–1.178)
	PM_10_	1.001 (0.999–1.003)	**1.002 (1.000–1.005)**	1.000 (0.999–1.001)
	PM_2.5_	1.000 (0.996–1.003)	1.001 (0.998–1.005)	0.999 (0.998–1.001)
Lag 6	O_3_	0.999 (0.994–1.004)	1.000 (0.992–1.008)	0.999 (0.997–1.000)
	NO	1.000 (0.999–1.002)	0.996 (0.975–1.018)	1.000 (0.993–1.006)
	NO_2_	1.001 (0.995–1.007)	1.013 (0.982–1.044)	1.017 (0.997–1.037)
	SO_2_	1.001 (0.987–1.033)	1.006 (0.976–1.038)	1.002 (0.994–1.009)
	CO	0.749 (0.364–1.539)	0.578 (0.228–1.468)	0.969 (0.796–1.178)
	PM_10_	0.999 (0.997–1.002)	0.998 (0.995–1.001)	1.000 (0.999–1.001)
	PM_2.5_	0.997 (0.993–1.001)	0.995 (0.991–1.000)	0.999 (0.998–1.001)
Lag 7	O_3_	0.998 (0.992–1.003)	0.999 (0.992–1.006)	0.999 (0.997–1.000)
	NO	**1.001 (1.000–1.003)**	1.010 (0.992–1.029)	1.000 (0.993–1.006)
	NO_2_	1.001 (0.995–1.007)	1.002 (0.977–1.028)	**1.020 (1.000–1.040)**
	SO_2_	1.010 (0.987–1.033)	1.006 (0.978–1.034)	1.002 (0.994–1.009)
	CO	0.988 (0.570–1.712)	1.008 (0.512–1.982)	0.969 (0.796–1.178)
	PM_10_	1.001 (0.999–1.004)	1.002 (0.999–1.004)	1.000 (0.999–1.001)
	PM_2.5_	1.000 (0.997–1.003)	1.001 (0.998–1.004)	0.999 (0.998–1.001)

Bold value indicates statistically significant.

**Table 3:** The increasedrisk (95% CIs) of DVT hospital admissions per 10 μg/m^3^increase in air pollutants in male and female subgroups.

Lag	Pollutant	Men			Women		
		Lag terms modeled one at time RR (95% CI)	Adjusted unconstrained DLM RR (95% CI)	Adjusted constrained DLM RR (95% CI)	Lag terms modeled one at time RR (95% CI)	Adjusted unconstrained DLM RR (95% CI)	Adjusted constrained DLM RR (95% CI)
Lag 0	O_3_	0.985 (0.969–1.001)	0.986 (0.970–1.002)	0.986 (0.970–1.002)	0.996 (0.986–1.005)	0.991 (0.980–1.003)	0.9943 (0.984–1.005)
	NO	**1.030 (1.007–1.055)**	**1.027 (1.002–1.052)**	**1.003 (1.001–1.005)**	1.005 (0.981–1.028)	1.002 (0.977–1.028)	**1.002 (1.000–1.004)**
	NO_2_	**1.061 (1.028–1.095)**	**1.066 (1.031–1.103)**	**1.049 (1.022–1.076)**	1.004 (0.973–1.035)	0.994 (0.961–1.029)	**1.026 (1.000–1.053)**
	SO_2_	1.030 (0.999–1.062)	1.028 (0.995–1.061)	1.018 (0.986–1.051)	0.993 (0.960–1.028)	0.977 (0.936–1.020)	0.972 (0.933–1.013)
	CO	1.432 (0.955–2.147)	1.599 (0.940–2.722)	1.641 (1.019–2.643)	1.059 (0.535–2.095)	1.136 (0.558–2.310)	1.126 (0.594–2.135)
	PM_10_	0.999 (0.995–1.003)	0.999 (0.994–1.003)	0.999 (0.995–1.003)	1.000 (0.997–1.004)	1.001 (0.998–1.004)	1.001 (0.998–1.004)
	PM_2.5_	1.000 (0.995–1.005)	0.999 (0.993–1.004)	0.999 (0.994–1.004)	**1.004 (1.001–1.008)**	**1.004 (1.001–1.008)**	**1.004 (1.001–1.007)**
Lag 1	O_3_	1.000 (0.995–1.005)	1.005 (0.992–1.019)	1.001 (0.998–1.004)	1.002 (0.997–1.006)	1.007 (0.997–1.018)	1.002 (0.999–1.004)
	NO	0.996 (0.979–1.013)	1.009 (0.979–1.039)	1.001 (0.999–1.004)	**1.014 (1.000–1.027)**	1.021 (0.996–1.047)	**1.002 (1.000–1.004)**
	NO_2_	0.981 (0.961–1.003)	0.970 (0.928–1.015)	1.003 (0.974–1.033)	1.015 (0.997–1.034)	**1.044 (1.002–1.087)**	**1.034 (1.009–1.060)**
	SO_2_	0.975 (0.949–1.001)	0.986 (0.938–1.036)	0.981 (0.943 1.021)	1.008 (0.977 1.039)	1.000 (0.956–1.046)	1.011 (0.990–1.033)
	CO	1.021 (0.489–2.132)	0.872 (0.392–1.938)	0.731 (0.383–1.397)	**1.333 (1.008–1.763)**	1.298 (0.746–2.258)	1.316 (0.793–2.184)
	PM_10_	1.001 (0.999–1.004)	**1.003 (1.001–1.006)**	1.003 (1.000–1.006)	0.996 (0.992–1.001)	0.997 (0.993–1.002)	0.997 (0.995 1.000)
	PM_2.5_	1.003 (0.999–1.006)	1.004 (0.999–1.008)	1.002 (0.999–1.005)	0.999 (0.997–1.002)	0.998 (0.993–1.003)	1.000 (0.995–1.004)
Lag 2	O_3_	1.001 (0.995–1.006)	0.999 (0.984–1.014)	1.001 (0.998–1.004)	1.001 (0.996–1.006)	0.996 (0.985–1.007)	1.002 (0.999–1.004)
	NO	0.996 (0.979–1.013)	0.992 (0.959–1.025)	0.998 (0.995–1.000)	**1.014 (1.000–1.027)**	1.014 (0.987–1.043)	1.001 (0.999–1.004)
	NO_2_	0.981 (0.961–1.003)	0.997 (0.952–1.045)	0.979 (0.949–1.009)	1.015 (0.997–1.034)	1.005 (0.963–1.049)	1.014 (0.987–1.041)
	SO_2_	0.975 (0.949–1.001)	0.973 (0.919–1.030)	0.951 (0.909–0.994)	1.020 (0.990–1.050)	1.019 (0.978–1.062)	1.011 (0.990–1.033)
	CO	0.380 (0.098–1.477)	0.505 (0.119–2.140)	0.731 (0.383–1.397)	**1.333 (1.008–1.763)**	1.5665 (0.936–2.622)	**1.550 (1.021–2.353)**
	PM_10_	1.001 (0.999–1.004)	1.000 (0.996–1.004)	1.000 (0.996–1.003)	0.997 (0.993–1.001)	0.999 (0.994–1.003)	0.997 (0.995–1.000)
	PM_2.5_	1.000 (0.995–1.004)	0.999 (0.994–1.005)	1.002 (0.999–1.005)	0.999 (0.997–1.002)	1.001 (0.997–1.005)	1.002 (0.998–1.005)
Lag 3	O_3_	0.988 (0.972–1.003)	0.993 (0.974–1.012)	0.999 (0.996–1.001)	0.998 (0.991–1.006)	1.000 (0.988–1.012)	1.002 (0.999–1.004)
	NO	0.996 (0.985–1.006)	0.969 (0.935–1.003)	0.997 (0.994–1.000)	1.003 (0.994–1.012)	0.983 (0.950–1.016)	0.999 (0.996–1.001)
	NO_2_	0.998 (0.989–1.008)	0.987 (0.941–1.035)	0.976 (0.946–1.007)	1.003 (0.995–1.012)	0.962 (0.919–1.007)	0.996 (0.968–1.024)
	SO_2_	0.997 (0.985–1.010)	0.978 (0.923–1.036)	0.949 (0.908–0.992)	1.021 (0.992–1.050)	1.007 (0.967–1.049)	1.005 (0.996–1.015)
	CO	0.671 (0.208–2.167)	0.922 (0.318–2.675)	0.901 (0.646–1.257)	1.041 (0.808–1.340)	0.715 (0.313–1.634)	1.049 (0.532–2.067)
	PM_10_	1.000 (0.998–1.001)	0.995 (0.989–1.000)	0.996 (0.991–1.001)	0.995-(0.990–1.000)	0.995 (0.990–1.001)	1.000 (0.998–1.001)
	PM_2.5_	0.999 (0.994–1.004)	0.998 (0.993–1.003)	0.999 (0.997–1.001)	1.000 (0.998–1.001)	0.999 (0.993–1.005)	0.998 (0.994–1.003)
Lag 4	O_3_	0.983 (0.967–1.000)	0.984 (0.964–1.005)	0.999 (0.996–1.001)	0.998 (0.990–1.006)	1.000 (0.988–1.013)	0.998 (0.995–1.001)
	NO	0.996 (0.985–1.006)	1.029 (0.997–1.061)	0.999 (0.996–1.002)	1.003 (0.994–1.012)	0.998 (0.967–1.029)	1.000 (0.998–1.003)
	NO_2_	0.998 (0.989–1.008)	1.012 (0.966–1.061)	0.974 (0.944–1.006)	1.003 (0.995–1.012)	1.026 (0.983–1.071)	1.024 (0.998–1.051)
	SO_2_	0.997 (0.985–1.010)	1.0091 (0.961–1.060)	0.971 (0.932–1.012)	1.020 (0.991–1.050)	1.004 (0.960–1.050)	1.005 (0.996–1.014968)
	CO	0.536 (0.147–1.950)	1.161 (0.337–4.000)	0.901 (0.646–1.257)	1.041 (0.808–1.340)	1.052 (0.576–1.920)	1.382 (0.860–2.222)
	PM_10_	1.000 (0.998–1.001)	0.999 (0.995–1.004)	0.998 (0.994–1.002)	0.999 (0.995–1.002)	1.000 (0.996–1.004)	1.000 (0.998–1.001)
	PM_2.5_	1.000 (0.996–1.004)	1.001 (0.996–1.006)	0.999 (0.997–1.001)	1.000 (0.998–1.001)	0.997 (0.991–1.003)	0.998 (0.993–1.003)
Lag 5	O_3_	0.998 (0.992–1.005)	0.999 (0.988–1.011)	0.999 (0.996–1.001)	0.996 (0.986–1.006)	0.999 (0.985–1.012)	0.998 (0.995–1.001)
	NO	0.996 (0.985–1.006)	0.953 (0.919–0.988)	0.996 (0.993–0.999)	1.003 (0.994–1.012)	**1.032 (1.005–1.060)**	**1.003 (1.001–1.005)**
	NO_2_	0.998 (0.989–1.008)	0.944 (0.900–0.990)	0.975 (0.944–1.007)	1.003 (0.995–1.012)	1.026 (0.985–1.070)	**1.035 (1.009–1.062)**
	SO_2_	0.997 (0.985–1.010)	0.958 (0.908–1.011)	0.973 (0.933–1.014)	1.024 (0.996–1.054)	1.020 (0.980–1.062)	1.005 (0.996–1.015)
	CO	0.363 (0.0934–1.408)	0.449 (0.098–2.056)	0.901 (0.646–1.257)	1.041 (0.808–1.340)	**2.194 (1.129–4.261)**	1.507 (0.975–2.328)
	PM_10_	1.000 (0.998–1.001)	1.003 (0.999–1.006)	1.002 (0.999–1.005)	1.000 (0.997–1.004)	1.002 (0.998–1.006)	1.000 (0.998–1.001)
	PM_2.5_	1.000 (0.995–1.004)	1.000 (0.995–1.006)	0.999 (0.997–1.001)	1.000 (0.998–1.001)	1.001 (0.996–1.006)	1.000 (0.995–1.004)
Lag 6	O_3_	1.001 (0.996–1.006)	1.004 (0.99–1.016)	0.999 (0.996–1.001)	0.990 (0.977–1.003)	0.992 (0.976–1.007)	0.998 (0.995–1.001)
	NO	0.996 (0.985–1.006)	1.013 (0.981–1.045)	1.000 (0.998–1.003)	1.003 (0.994–1.012)	0.982 (0.953–1.012)	1.000 (0.998–1.003)
	NO_2_	0.998 (0.989–1.008)	1.033 (0.988–1.080)	1.016 (0.987–1.046)	1.003 (0.995–1.012)	0.994 (0.952–1.038)	1.018 (0.991–1.046)
	SO_2_	0.997 (0.985–1.010)	**1.042 (1.000–1.086)**	1.016 (0.982–1.050)	1.005 (0.974–1.038)	0.971 (0.926–1.019)	1.005 (0.996–1.015)
	CO	0.758 (0.261–2.206)	1.006 (0.371–2.728)	0.901 (0.646–1.257)	1.041 (0.808–1.340)	0.370 (0.091–1.504)	0.703 (0.258–1.916)
	PM_10_	1.000 (0.998–1.001)	0.999 (0.995–1.003)	1.000 (0.996–1.004)	0.999 (0.995–1.003)	0.997 (0.993–1.002)	1.000 (0.998–1.001)
	PM_2.5_	0.999 (0.994–1.004)	0.999 (0.993–1.004)	0.999 (0.997–1.001)	1.000 (0.998–1.001)	0.993 (0.986–1.000)	0.994 (0.988–1.001)
Lag 7	O_3_	0.998 (0.991–1.005)	0.996 (0.984–1.009)	0.999 (0.996–1.001)	0.997 (0.988–1.006)	1.001 (0.993–1.010)	0.998 (0.995–1.001)
	NO	0.996 (0.985–1.006)	1.009 (0.982–1.036)	**1.002 (1.000–1.004)**	1.003 (0.994–1.012)	1.013 (0.986–1.041)	1.001 (0.998–1.003)
	NO_2_	0.998 (0.989–1.0080)	1.012 (0.975–1.050)	**1.033 (1.004–1.062)**	1.003 (0.995–1.012)	0.993 (0.957–1.030)	1.009 (0.981–1.037)
	SO_2_	0.997 (0.985–1.010)	0.989 (0.946–1.033)	1.003 (0.967–1.040)	1.017 (0.987–1.048)	1.023 (0.986–1.062)	1.005 (0.996–1.015)
	CO	1.074 (0.544–2.121)	1.114 (0.498–2.490)	0.901 (0.646–1.257)	1.041 (0.808–1.340)	0.881 (0.292–2.657)	0.845 (0.347–2.056)
	PM_10_	1.000 (0.998–1.001)	1.001 (0.998–1.005)	1.001 (0.998–1.004)	1.002 (0.999–1.005)	1.002 (0.999–1.005)	1.000 (0.998–1.001)
	PM_2.5_	0.996 (0.990–1.002)	0.997-(0.990–1.003)	0.999 (0.997–1.001)	1.000 (0.998–1.001)	**1.004 (1.000–1.008)**	1.002 (0.999–1.005)

Bold value indicates statistically significant.

**Table 4:** The increasedrisk (95% CIs) of DVT hospital admissions per 10 μg/m^3^increase in air pollutants according in the age subgroups.

≤60years					>60 years		
Lag	Pollutant	Lag terms modeled one at time RR (95% CI)	Adjusted unconstrained DLM RR (95% CI)	Adjusted constrained DLM RR (95% CI)	Lag terms modeled one at time RR (95% CI)	Adjusted unconstrained DLM RR (95% CI)	Adjusted constrained DLM RR (95% CI)
Lag 0	O_3_	0.994 (0.984–1.005)	0.993 (0.982–1.004)	0.994 (0.984–1.005)	0.987 (0.971–1.003)	0.980 (0.964–0.997)	0.986 (0.970–1.002)
	NO	1.002 (0.999–1.004)	1.010 (0.984–1.036)	1.010 (0.986–1.035)	**1.025 (1.003–1.048)**	1.020 (0.996–1.045)	**1.003 (1.001–1.005)**
	NO_2_	1.014 (0.988–1.042)	1.011 (0.976–1.047)	1.007 (0.975–1.041)	**1.059 (1.027–1.091)**	**1.051 (1.016–1.087)**	**1.062 (1.036–1.089)**
	SO_2_	1.006 (0.975–1.038)	1.014 (0.977–1.053)	1.008 (0.972–1.046)	1.005 (0.970–1.041)	1.002 (0.964–1.041)	1.004 (0.968–1.041)
	CO	1.227 (0.817–1.842)	1.187 (0.696–2.023)	1.212 (0.783–1.874)	1.840 (0.798–4.243)	2.229 (0.850–5.843)	1.870 (0.733–4.768)
	PM_10_	1.001 (0.998–1.004)	1.001 (0.997–1.004)	1.001 (0.998–1.004)	0.998 (0.994–1.002)	0.998 (0.994–1.002)	0.998 (0.994–1.002)
	PM_2.5_	**1.005 (1.002–1.009)**	**1.005 (1.002–1.009)**	**1.004 (1.002–1.007)**	0.999 (0.993–1.004)	0.997 (0.991–1.002)	0.996 (0.991–1.002)
Lag 1	O_3_	1.001 (0.997–1.005)	1.005 (0.995–1.015)	1.001 (0.999–1.003)	1.000 (0.995–1.006)	1.013 (0.999–1.028)	1.001 (0.998–1.004)
	NO	1.000 (0.998–1.003)	1.001 (0.972–1.030)	1.000 (0.985–1.016)	1.010 (0.996–1.025)	**1.027 (1.000–1.054)**	**1.003 (1.001–1.005)**
	NO_2_	0.998 (0.970–1.025)	0.9889 (0.948–1.032)	0.996 (0.977–1.016)	1.001 (0.981–1.021)	1.027 (0.984–1.072)	**1.046 (1.018–1.074)**
	SO_2_	0.990 (0.957–1.025)	0.975 (0.927–1.025)	0.994 (0.971–1.018)	1.002 (0.967–1.039)	1.006 (0.965–1.050)	0.994 (0.970–1.018)
	CO	1.173 (0.757–1.818)	1.187 (0.696–2.023)	1.112 (0.821–1.504)	1.050 (0.312–3.534)	0.641 (0.133–3.081)	1.126 (0.510–2.487)
	PM_10_	0.999 (0.997–1.002)	1.002 (0.999–1.005)	1.001 (0.999–1.004)	0.998 (0.994–1.003)	0.999 (0.995–1.003)	1.000 (0.997–1.002)
	PM_2.5_	0.999 (0.997–1.002)	1.001 (0.997–1.006)	1.001 (0.998–1.005)	1.001 (0.997–1.005)	1.000 (0.995–1.005)	1.001 (0.998–1.004)
Lag 2	O_3_	1.002 (0.997–1.006)	0.998 (0.987–1.009)	1.001 (0.999–1.003)	1.000 (0.993–1.007)	0.991 (0.976–1.006)	1.001 (0.998–1.004)
	NO	0.999 (0.997–1.002)	1.007 (0.977–1.038)	1.000 (0.985–1.016)	1.010 (0.996–1.025)	1.002 (0.972–1.033)	1.000 (0.998–1.003)
	NO_2_	0.992 (0.965–1.021)	1.008 (0.965–1.052)	0.996 (0.977–1.016)	1.001 (0.981–1.021)	0.994 (0.949–1.042)	1.003 (0.975–1.033)
	SO_2_	1.002 (0.971–1.035)	1.000 (0.953–1.048)	0.994 (0.971–1.018)	0.977 (0.937–1.018)	0.998 (0.953–1.046)	0.994 (0.970–1.018)
	CO	1.138 (0.711–1.821)	1.081 (0.618–1.891)	1.112 (0.821–1.504)	1.340 (0.466–3.851)	2.491 (0.786–7.899)	1.126 (0.510–2.487)
	PM_10_	0.999 (0.997–1.002)	0.996 (0.992–1.001)	0.997 (0.992–1.001)	1.000 (0.996–1.004)	1.002 (0.999–1.006)	1.000 (0.997–1.002)
	PM_2.5_	0.999 (0.997–1.002)	0.998 (0.994–1.003)	1.001 (0.997–1.004)	1.001 (0.997–1.005)	1.002 (0.997–1.007)	1.001 (0.998–1.004)
Lag 3	O_3_	0.997 (0.989–1.005)	1.001 (0.989–1.013)	0.998 (0.996–1.001)	0.991 (0.976–1.006)	0.992 (0.973–1.012)	0.999 (0.996–1.001)
	NO	0.998 (0.996–1.001)	0.974 (0.942–1.008)	1.000 (0.991–1.009)	1.000 (0.991–1.010)	0.976 (0.943–1.011)	0.998 (0.995–1.000)
	NO_2_	0.988 (0.960–1.016)	0.983 (0.941–1.028)	1.002 (0.993–1.010)	1.001 (0.992–1.010)	0.962 (0.916–1.011)	0.985 (0.955–1.016)
	SO_2_	1.015 (0.985–1.044)	1.029 (0.987–1.072)	1.006 (0.996–1.016)	0.956 (0.914–1.000)	0.949 (0.897–1.004)	0.996 (0.984–1.008)
	CO	0.994 (0.555–1.780)	0.902 (0.467–1.742)	0.979 (0.778–1.232)	0.473 (0.107–2.080)	0.317 (0.051–1.985)	0.949 (0.624–1.443)
	PM_10_	1.000 (0.998–1.001)	0.998 (0.994–1.003)	0.997 (0.993–1.001)	0.993 (0.987–0.999)	0.989 (0.982–0.996)	1.000 (0.998–1.001)
	PM_2.5_	0.999 (0.997–1.001)	0.999 (0.994–1.004)	0.998 (0.993–1.003)	0.999 (0.994–1.004)	0.999 (0.994–1.004)	1.000 (0.999–1.002)
Lag 4	O_3_	0.994 (0.984–1.005)	0.994 (0.980–1.008)	0.998 (0.996–1.001)	0.992 (0.978–1.006)	0.999 (0.982–1.016)	0.999 (0.996–1.001)
	NO	1.001 (0.998–1.003)	1.029 (0.999–1.060)	1.000 (0.991–1.010)	1.000 (0.991–1.010)	0.998 (0.966–1.031)	0.999 (0.996–1.002)
	NO_2_	1.001 (0.973–1.029)	1.028 (0.984–1.074)	1.002 (0.993–1.010)	1.001 (0.992–1.010)	1.010 (0.963–1.059)	1.004 (0.974–1.034)
	SO_2_	1.013 (0.983–1.044)	1.013 (0.972–1.056)	1.006 (0.996–1.016)	0.982 (0.943–1.022)	0.996 (0.949–1.047)	0.996 (0.984–1.008)
	CO	1.147 (0.718–1.833)	1.080 (0.583–2.000)	0.979 (0.778–1.232)	0.696 (0.173–2.805)	0.800 (0.157–4.061)	0.949 (0.624–1.44)
	PM_10_	1.000 (0.998–1.001)	0.998 (0.993–1.002)	0.997 (0.993–1.001)	1.000 (0.996–1.004)	1.002 (0.998–1.006)	1.000 (0.998–1.001)
	PM_2.5_	0.999 (0.997–1.001)	0.997 (0.991–1.003)	0.997 (0.992–1.002)	1.001 (0.997–1.005)	1.001 (0.996–1.006)	1.000 (0.999–1.002)
Lag 5	O_3_	0.997 (0.989–1.005)	1.004 (0.991–1.017)	0.998 (0.996–1.001)	0.999 (0.991–1.006)	0.993 (0.979–1.006)	0.999 (0.996–1.001)
	NO	0.999 (0.997–1.002)	0.987 (0.955–1.019)	1.000 (0.991–1.009)	1.000 (0.991–1.001)	1.011 (0.981–1.040)	1.001 (0.998–1.003)
	NO_2_	1.001 (0.973–1.029)	0.972 (0.930–1.015)	1.002 (0.993–1.010)	1.001 (0.992–1.010)	1.010 (0.965–1.057)	1.019 (0.990–1.049)
	SO_2_	1.004 (0.973–1.036)	0.981 (0.935–1.028)	1.006 (0.996–1.016)	1.006 (0.970–1.043)	1.004 (0.960–1.050)	0.996 (0.984–1.008)
	CO	1.102 (0.661–1.837)	1.292 (0.599–2.788)	0.979 (0.778–1.232)	1.286 (0.427–3.869)	1.668 (0.529–5.260)	0.949 (0.624–1.443)
	PM_10_	1.000 (0.998–1.001)	1.002 (0.999–1.006)	1.000 (0.997–1.003)	1.001 (0.998–1.005)	1.002 (0.998–1.005)	1.000 (0.998–1.001)
	PM_2.5_	0.999 (0.997–1.001)	1.002 (0.997–1.007)	0.999 (0.994–1.003)	1.001 (0.997–1.005)	1.000 (0.995–1.006)	1.000 (0.999–1.002)
Lag 6	O_3_	0.994 (0.983–1.005)	0.993 (0.980–1.006)	0.998 (0.996–1.001)	1.001 (0.996–1.006)	1.011 (0.997–1.025)	0.999 (0.996–1.001)
	NO	1.000 (0.998–1.003)	1.000 (0.969–1.032)	1.000 (0.991–1.009)	1.000 (0.991–1.010)	0.991 (0.962–1.021)	1.000 (0.998–1.003)
	NO_2_	1.015 (0.989–1.043)	1.019 (0.976–1.063)	1.002 (0.993–1.010)	1.001 (0.992–1.010)	1.000 (0.957–1.046)	1.019 (0.990–1.049)
	SO_2_	1.011 (0.981–1.042)	1.012 (0.971–1.060)	1.006 (0.996–1.016)	1.010 (0.975–1.046)	0.997 (0.955–1.041)	0.996 (0.984–1.008)
	CO	0.614 (0.230–1.638)	0.561 (0.164–1.921)	0.979 (0.778–1.232)	1.010 (0.291–3.497)	0.643 (0.127–3.254)	0.949 (0.624–1.443)
	PM_10_	1.000 (0.998–1.001)	0.994 (0.989–0.999)	0.997 (0.993–1.001)	1.002 (0.998–1.005)	1.003 (0.999–1.006)	1.000 (0.998–1.001)
	PM_2.5_	0.999 (0.997–1.001)	0.990 (0.982–0.998)	0.992 (0.985–0.998)	1.001 (0.997–1.006)	1.000 (0.995–1.005)	1.000 (0.999–1.002)
Lag 7	O_3_	0.998 (0.991–1.006)	1.001 (0.995–1.008)	0.998 (0.996–1.001)	0.997 (0.987–1.006)	0.989 (0.975–1.003)	0.999 (0.996–1.001)
	NO	1.001 (0.999–1.003)	1.004 (0.976–1.032)	1.000 (0.991–1.009)	1.000 (0.991–1.010)	1.021 (0.995–1.048)	1.002 (0.999–1.004)
	NO_2_	1.019 (0.992–1.046)	1.001 (0.965–1.038)	1.002 (0.993–1.010)	1.001 (0.992–1.010)	1.012 (0.976–1.050)	1.023 (0.995–1.052)
	SO_2_	1.008 (0.978–1.039)	0.998 (0.959–1.039)	1.006 (0.996–1.016)	1.015 (0.981–1.050)	1.018 (0.979–1.058)	0.996 (0.984–1.008)
	CO	0.715 (0.293–1.747)	0.818 (0.267–2.508)	0.979 (0.778–1.232)	1.765 (0.761–4.095)	1.536 (0.607–3.888)	0.949 (0.624–1.443)
	PM_10_	1.000 (0.998–1.001)	**1.005 (1.002–1.007)**	**1.003 (1.001–1.006)**	0.998 (0.994–1.003)	0.997 (0.992–1.001)	1.000 (0.998–1.001)
	PM_2.5_	0.999 (0.997–1.001)	1.002 (0.998–1.007)	1.000 (0.995–1.004)	1.001 (0.997–1.005)	1.001 (0.996–1.005)	1.000 (0.999–1.002)

Bold value indicates statistically significant.

The results showed that there was a significant relation between NO_2_ and hospital admissions for DVT in total (Table 2), males, females (Table 3), and people >60 years of age (Table 4). Accordingly, for increase inNO_2_ concentrations by 10 μg/m^3^, the risk of hospital admissions for DVT in the total population, males, females, and people >60 years of age increased by 2.8, 6.6, 4.4, and 5.1% in adjusted unconstrained modelson0, 0, 1, and 0 day lags (Tables 2, 3, and 4). The effect of NO_2_ wasmainly with short (0–1 day) lags.

A significant association was seenbetween SO_2_ and hospital admissions for DVT in the male population; and for a 10μg/m^3^increasein SO_2_ concentrations, the risk of hospital admissions for DVT in the male population increased by 4.2% aftera 6-day lagin adjusted unconstrained models (Table 3). There was alsoa significant associationbetween CO and hospital admissions for DVT in the female population; and for a 10 μg/m^3^ increase inCO concentration, the risk of hospital admissions for DVT increased by 119.4% after a5-day lagin adjusted unconstrained models (Table 3).

There were alsosignificant relations between PM_10_ and hospital admissions for DVT in the male and ≤60 years old population; andfor 10 μg/m^3^increase inPM_10_ concentration, the risk of hospital admissions for DVT in the male population and the ≤60 years old population increased by 0.3 and 0.5% after 1 and 7-day lagsin adjusted unconstrained models (Tables 3 and 4).

Finally, there was a significant relation between PM_2.5_ and hospital admissions for DVT in the female and ≤60years old population. Accordingly, for each 10μg/m^3^increase in PM_2.5_ concentration, the risk of hospital admissions for DVT in the female and ≤60 years old population increased by 0.4 and 0.5% on the same dayin adjusted unconstrained models (Tables 3 and 4). The effect of NO2 was mainly with short (0 day) lags.

O_3_ exposure and total hospital admissions due to DVT were significantly associated in lag 1 and 2 ofadjusted constrained models (Table 2).

## 4 Discussion

The purpose of this study was to determine the relation between air pollutants and hospital admissions due to DVT.

The results of this study showed that there was a significant relation between exposure to NO and NO_2_and hospital admissions due to DVT in some subpopulations and time lags.

In this regard, in a study conducted by Spiezia et al. (2014) on patients with thrombosis in the city of Padua, exposure to environmental pollutants including PM_10_, NOx, benzene, benzoapyrene, cadmium, and lead caused a significant increase in the risk of unprovoked PE(Spiezia et al., 2014).

A study by De Marco et al. (2019) in Italy, predicted that PM_10_ and NO_2_will be related tocardiovascular, respiratory, and early mortality of 58,000 people in Europe in 2055. Theyalso stated that in 2015, abouthalf a million sudden deaths occurred in European Union member states due to exposure to O_3_, PM_10_, and NO_2_(De Marco et al., 2019).

Moshammer et al. (2019) studied the mechanism of the effect of nitrous oxide on the respiratory system (the first level of contact with pollutants) and the cardiovascular system of volunteers walkingin parks or beside busyroads and showedPM_2.5_, PM_1_ and NO adversely affect lung and cardiovascular function (Moshammer et al., 2019).

The results of this study showed that there was a direct and significant relation between exposure to PM_10_ and hospital admissions due to DVT in the male and ≤60 years old population. There was also a direct and significant relation between exposure to PM_2.5_ and hospital admissions due to DVT in the female and ≤60 years old population. The reason that more adverse effects are being seen in the under 60 population might be the fact that this population is more active, and spends more time outdoors than the over 60 population which is mainly retired and goes out occasionally or for leisure.

Xu et al. (2019) in Beijing and Chen et al. (2017) in Taiwan showed that air pollution canaffect the human cardiovascular system and cause long-term inflammation. Signorelli et al. (2017) stated that air pollution and inhalation of PM_10_, PM_2.5_ and PM_1_ play an important role in DVT, PE, and VTE (Signorelli et al., 2017). Emmerechts et al. (2012) studied the relation between PM and VTE in Belgium and found that VTE was associated with long-term exposure to air pollution (Emmerechts et al., 2012). A study by Kloog et al. (2015) included a wide geographical range ofnortheastern US states, and showed that PM_2.5_concentration was associated with increased DVT and PEin individuals aged 65 and older. The researchers in this study commented that air pollution is associated with activation of inflammatory pathways, reactive oxygen species generation, endothelial damage, arterial vascular mobility dysfunction and changes in blood coagulation factors (Kloog et al., 2015). Finally, a review conducted by Franchini and Mannucci (2011) on more than 100 studies, stated that according to the results of empirical, clinical and epidemiological studies, air pollution seems to be effective on cardiovascular, cerebrovascular, and coronary artery diseasesand PM can be a serious risk for VTE. Moreover, this review found that these effects are greater in areas with higher levels of air pollution, such as high traffic areas (Franchini and Mannucci, 2011).

In this study, there was a significant and direct correlation between exposure to SO_2_ and hospital admissions due to DVT in the male population. Studies have shown a relation between SO_2_ and the risk of cardiovascular and respiratory diseases morbidity. Kim et al. (2019) showed that SO_2_hada positive impact on cardiovascular and respiratory diseases in South Korea (Kim et al., 2019). In Kuala Lumpur, Malaysia increased concentrations of ambient SO_2_ and NO_2_ had the greatest impact on the rate of hospitalization of patients due to cardiovascular and respiratory diseases (Tajudin et al., 2019). And in the Netherlands, exposure to ozone (with 1 day lag) and SO_2_ (7-day mean) were associated with increased embolism and thrombosis mortality (Hoek et al., 2001).

The results of this study showed that there was a direct relation between exposure to CO and hospital admissions due to DVT in the female population. Accordingly, Chung et al., showed that the risk of DVT and PE development in subjects exposed to CO was significantly higher in Taiwan (Chung et al., 2015). We did not find a reason for women being more prone for developing DVT in exposure to CO. However, researchers have mentioned certain individuals, such as children, the elderly, pregnant women, and people with chronic diseases may be more susceptible to increased levels of CO (Townsend and Maynard, 2002). Also obesity and being inactive (Samama, 2000) which is more common among women than men in Iran (Rahmani et al., 2015) and using birth control pills (oral contraceptives) or hormone replacement therapy can increase coagulability (Speroff, 1996).

One of the strengths of this study was that this research was the first study done about this topic in one of the most polluted cities in the world, and in gender and age subgroups. Moreover, since this study was a time-series study, individual demographic variables such as age, socioeconomic status, employment, smoking, and daily habits remained relatively constant during the study in the population. One of the limitations of this study was the use of aggregated data, which prevents the generalization of results to individual levels. Also, DVT can be managed outside the hospital setting and therefore only patients with severe DVT or other comorbidities are admitted to hospitals and were included in our study.

## 5 Conclusion

The results of this study showed that short-term exposure to ambient pollutants such as SO_2_, PM_10_, PM_2.5_, NO, NO_2_, and CO is probably associated with increased hospital admissions due to DVT. However, further studies are required to investigate whether direct interventions through industry and government policy may alter the impact of specific pollutants in order to alter the incidence of DVT and other identified health complications.

## Declarations

### Author contribution statement

S. Borsi and H. Raji: Conceived and designed the experiments.

M. Dastoorpoor and N. Khanjani: Analyzed and interpreted the data; Wrote the paper.

Z. Sekhavatpour: Analyzed and interpreted the data.

H. Nejad: Contributed reagents, materials, analysis tools or data.

A. Riahi: Contributed reagents, materials, analysis tools or data; Wrote the paper.

### Funding statement

This work was supported by 10.13039/501100005001Ahvaz Jundishapur University of Medical Sciences, Ahvaz, Iran (Grant No: APRD-9705).

### Competing interest statement

The authors declare no conflict of interest.

### Additional information

No additional information is available for this paper.
